# Thermal-Enhanced bri1-301 Instability Reveals a Plasma Membrane Protein Quality Control System in Plants

**DOI:** 10.3389/fpls.2018.01620

**Published:** 2018-11-06

**Authors:** Minghui Lv, Meizhen Li, Weiyue Chen, Yanze Wang, Chao Sun, Hongju Yin, Kai He, Jia Li

**Affiliations:** Ministry of Education Key Laboratory of Cell Activities and Stress Adaptation, School of Life Sciences, Lanzhou University, Lanzhou, China

**Keywords:** Arabidopsis, BRI1, protein folding, endoplasmic reticulum quality control, plasma membrane quality control

## Abstract

Brassinosteroids (BRs) are essential phytohormones mainly perceived by a single-pass transmembrane receptor-like protein kinase (RLK), BRASSINOSTEROID INSENSITIVE 1 (BRI1). *bri1-5* and *bri1-9*, two distinct mutants with point mutations in the extracellular domain of BRI1, show weak defective phenotypes. Previous studies indicated that bri1-5 and bri1-9 mutated proteins can be recognized and eliminated via an endoplasmic reticulum quality control (ERQC) mechanism. Most of these two proteins, therefore, cannot reach their destination, plasma membrane. Here, we report our functional characterization of bri1-301, another BRI1 mutant protein with an amino acid substitution in the cytoplasmic kinase domain. bri1-301 is a partially functional BR receptor with significantly decreased protein abundance. Interestingly, protein stability and subcellular localization of bri1-301 are temperature-sensitive. At 22°C, an optimal temperature for indoor Arabidopsis growth, *bri1-301* shows a weak defective phenotype. At a lower temperature condition such as 18°C, *bri1-301* exhibits subtle morphological defects. At a higher temperature condition such as 28°C, on the other hand, *bri1-301* displays an extremely severe phenotype reminiscent to that of a null *bri1* mutant due to greatly increased bri1-301 internalization and degradation. Our detailed analyses suggest that bri1-301 stability is controlled by ERQC and plasma membrane quality control (PMQC) systems. Since PMQC has not been well studied in plants, *bri1-301* can be used as a model mutant for future genetic dissection of this critical process.

## Introduction

Brassinosteroids (BRs) are a group of polyhydroxylated phytohormones widely identified in plant kingdom. BRs regulate various physiological processes during growth and development (Grove et al., 1979; Clouse and Sasse, 1998; Zhang et al., 2009; Vilarrasa-Blasi et al., 2014; Lee et al., 2015). Arabidopsis mutants with defects in BR biosynthesis, perception, or signal transduction usually display similar phenotypic abnormalities including dwarfism, dark green and compact rosette leaves, delayed senescence, reduced male fertility, and de-etiolation in the dark (Clouse et al., 1996; Szekeres et al., 1996; Li et al., 2001).

Brassinosteroids are perceived by a protein complex consisting of BRASSINOSTEROID INSENSITIVE1 (BRI1) and BRI1-ASSOCIATED RECEPTOR KINASE1 (BAK1), both of which are single-pass transmembrane leucine-rich repeat receptor-like protein kinases (LRR-RLKs) (Li and Chory, 1997; Li et al., 2002; Nam and Li, 2002). Binding of BRs to the extracellular domain of BRI1 relieves its kinase domain from an inhibitory state caused by both the interference of its C-terminal tail as well as an inhibitory binding protein, BRI1 KINASE INHIBITOR 1 (BKI1) (Wang et al., 2005b; Wang and Chory, 2006; Jaillais et al., 2011). Physical interaction between BRs and the extracellular domain of BRI1 generates a docking platform for the recruitment of the co-receptor, BAK1 (Hothorn et al., 2011; She et al., 2011; He et al., 2013; Santiago et al., 2013). Only when BRI1-BR-BAK1 is formed, both BRI1 and BAK1 are activated through a reciprocal transphosphorylation mechanism (Wang et al., 2008). Fully activated BRI1 then triggers downstream signaling cascade predominantly via protein phosphorylation and dephosphorylation (Li and Nam, 2002; Mora-Garcia et al., 2004; Tang et al., 2008; Kim et al., 2009, 2011; Tang et al., 2011). Transcription factors, including well-characterized BZR1 and BES1, are subsequently activated and thousands of their target genes are transcriptionally regulated (Wang et al., 2002; Yin et al., 2002; He et al., 2005; Sun et al., 2010; Yu et al., 2011).

As the major BR receptor, BRI1 has been extensively studied and over 30 unique *bri1* mutant alleles have been identified through a number of independent genetic screens (Clouse, 1996; Li and Chory, 1997; Noguchi et al., 1999; Friedrichsen et al., 2000; Xu et al., 2008; Belkhadir et al., 2010; Shang et al., 2011; Gou et al., 2012; Sun et al., 2017). Severe phenotypes of *bri1* null alleles revealed the crucial roles of BRI1 on growth and development (Clouse, 1996; Li and Chory, 1997; Friedrichsen et al., 2000; Gou et al., 2012). Meanwhile, studies on *bri1* weak alleles contributed to our better understanding of several BRI1-associated cellular processes. For example, *bri1-9* is a semi-dwarfed *bri1* mutant with an S662F substitution within the BR binding domain of BRI1. It was therefore predicted that this mutation might have disrupted bri1-9 to bind its ligand (Friedrichsen et al., 2000). A genetic suppressor screen discovered that *bri1-9* can be suppressed by a loss-of-function mutant of *EBS1*, which encodes a UDP-glucose:glycoprotein glucosyltransferase essential to endoplasmic reticulum-mediated protein quality-control (ERQC) (Jin et al., 2007). ERQC is a highly conserved mechanism that monitors the protein folding process, allowing export of only correctly folded proteins, but retaining misfolded proteins (e.g., bri1-9) (Smith et al., 2011). Loss-of-function mutant of EBS1 in *bri1-9* reduces ERQC stringency and allows misfolded bri1-9 protein to export to the cell surface, resulting in a phenotypic suppression of the mutant (Jin et al., 2007). The wild-type-like morphology of *ebs1-1 bri1-9* suggests that the defective phenotype of *bri1-9* is likely caused by lack of plasma-membrane-localized BR receptor due to ER retention rather than disrupted BR binding of bri1-9 (Jin et al., 2007). Similar to bri1-9, another extracellular-domain mutant of BRI1, bri1-5, is also retained in the ER by the ERQC mechanism and is degraded by the ERAD process (Noguchi et al., 1999; Hong et al., 2008).

*bri1-301* is a weak *bri1* allele that contains two tandem nucleotide mutations but only one single amino acid substitution of G989I within the VIa kinase subdomain (Vert et al., 2005; Xu et al., 2008). *In vitro* autophosphorylation analysis indicated that bri1-301 does not show any kinase activity. In addition, bri1-301 is unable to phosphorylate BAK1, the co-receptor and also one of the known substrates of BRI1 (Xu et al., 2008). Nevertheless, molecular mechanisms explaining the weak phenotype of *bri1-301* have never been elucidated. Our initial interest is to investigate why such a kinase inactive *bri1* mutant shows a weak instead of a strong phenotype. Is the kinase activity truly non-essential for the biological functions of BRI1? If it is true, how can we explain the severe phenotypes of all other kinase-dead *bri1* mutants identified so far?

Here, we show that bri1-301 possesses kinase-activity *in vivo*, although greatly reduced. More interestingly, we report that the morphological severity of *bri1-301* and the protein accumulation of bri1-301 are temperature-dependent. In addition, the PM-localization of bri1-301 is normal at 22°C but is greatly disrupted at 28°C. Our results suggest a possible PMQC mechanism which is involved in recognition and removal of non-native membrane proteins such as bri1-301.

## Materials and Methods

### Plant Materials, Growth Conditions, and Phenotypic Analysis

*Arabidopsis thaliana* mutants used in this study include *bri1-301*, *det2-1*, *cpd91* (Du et al., 2016), *bri1-705* (Sun et al., 2017), *bin2-1*, *cpd* (Du et al., 2016) and *bri1-701*, all of which are Col-0 background. Plants were grown under long-day light condition (16 h light and 8 h dark) or darkness at 18°C, 22°C, and 28°C, respectively.

For seedlings, 7-day-old light-grown and 4-day-old dark-grown seedlings on 1/2 MS plates were photographed. For adults, the rosettes and leaves were photographed at indicated time points. All measurements were carried out using ImageJ^1^. Three independent biological replicates were carried out and at least 20 seedlings were used for each measurement. The statistical significance was evaluated by Student’s *t*-test.

### BL Treatment

After surface sterilization with 30% (v/v) bleach for 10 min and washing several times with sterile deionized water, seeds were sown on 1/2 MS medium plates containing 0.8% (w/v) agar and 1% (w/v) sucrose supplemented with or without 1 μM 24-epibrassinolide (Sigma). The plates were vernalized for 3 days at 4°C and then were transferred to growth chamber. Photos were taken at the indicated time points and the root length were measured as described above. Three independent biological replicates were carried out and the statistical significance was evaluated by Student’s *t*-test.

### RNA Extraction and Quantitative Real-Time PCR

11/9/7-day-old seedlings of Col-0 and *bri1-301* grown on 1/2 MS plates at 18°C/22°C/28°C were treated with or without 1 μM 24-epibrassinolide in deionized water for 1.5 h at same temperature condition. RNA extraction, reverse transcription and quantitative real-time PCR were performed as previously described (Zhao et al., 2016). Primers used in this study are listed in Supplementary Table S1. Three independent biological replicates were carried out for all the quantitative analyses.

### Vector Construction and Transgenic Plant Generation

The full-length *BAK1*, *BRI1-301* and other mutated *BRI1* coding sequence were cloned into destination vector *pBIB-35S-GWR-FLAG* (Gou et al., 2010) through two-step Gateway technology (Invitrogen, Life Technologies). The constructs were then transformed into Col-0, *bri1-301*, and *bri1-701* background through floral dip method (Clough and Bent, 1998). The *35S::BRI1-FLAG* and *35S::BAK1-GFP* transgenic plants were described in a previous report (Zhao et al., 2016).

### Protein Extraction, Immunoprecipitation, and Western Blot Analysis

For BES1 and BRI1/bri1 detection, the materials were prepared as described in RNA analysis. Total proteins were isolated with extraction buffer as described previously (Zhou et al., 2017). The lysates were centrifuged at 16,000 ×*g* for 10 min at 4°C after vortexing and the supernatant was then separated on a 12% (for BES1) and 7% (for BRI1) Bis-Tris SDS-PAGE gel and analyzed by immunoblotting with anti-BES1 serum, anti-BRI1 antibody (Agrisera) or anti-FLAG (Sigma) and anti-Tubulin (Sigma). The gray values of signal bands were measured by ImageJ 1.4.3 software (see footnote 1).

For the *in vivo* phosphorylation assay, transgenic plants were grown on 1/2 MS plates for 11/9/7 days at 18°C/22°C/28°C and then were treated with or without 1 μM BL for 1.5 h at the same temperature. After grinding in liquid nitrogen, the powder was lysed with extraction buffer (10 mM HEPES (pH 7.5), 100 mM NaCl, 1 mM EDTA, 10% glycerol, 0.5% Triton X-100 and 1:100 protease inhibitor cocktail from Roche). After vortexing vigorously for 30 s, the samples were centrifuged at 16,000 ×*g* for 10 min at 4°C, and the supernatant was then incubated with anti-FLAG (Sigma) agarose beads for 2 h at 4°C with gentle shaking. The immunoprecipitated proteins were separated on a 7% Bis-Tris SDS-PAGE gel and were analyzed by immunoblotting with anti-FLAG (Abmart), or anti-Pi-Thr antibody (Cell Signaling Technology).

### Confocal Microscopy

Five-day-old *pBRI1::BRI1-GFP* and *pBRI1::bri1-301-GFP* transgenic seedlings grown at 22°C and 28°C were used for imaging. For protein abundance observation, seedlings were directly mounted and scanned. For subcellular localization observation, seedlings were treated with propidium iodide (aqueous solution, 10 μg/mL) for 10 min, then were mounted in water and immediately observed under a Zeiss LSM 510 confocal microscope. The BRI1-GFP, bri1-301-GFP and PI were excited with 488-nm wavelength. The fluorescence emissions were detected with spectral detector set BP 505-560. Laser intensity and detection settings were kept constant except a higher detector gain value for bri1-301-GFP signal during its subcellular localization study.

### Endo H Treatment

11/9/7-day-old Col-0, *bri1-301* and *bri1-5* seedlings grown on 1/2 MS agar plates at 18°C/22°C/28°C were harvested and the total protein extracts were prepared as described previously (Hong et al., 2012). Briefly, seedlings were ground in liquid N_2_, dissolved in 2× SDS buffer containing 125 mM Tris (pH 6.8), 4% (w/v) SDS, 20% (v/v) glycerol, 200 mM DTT, 0.02% (w/v) bromophenol blue. After boiling and centrifugation, supernatants were incubated with or without 1,000 U of Endo H in 1× G5 buffer (New England Biolabs) for 1 h at 37°C. The Western blot analyses were performed as above.

## Results

### Phenotypic Severity of *bri1-301* Is Temperature-Dependent

**FIGURE 1:**
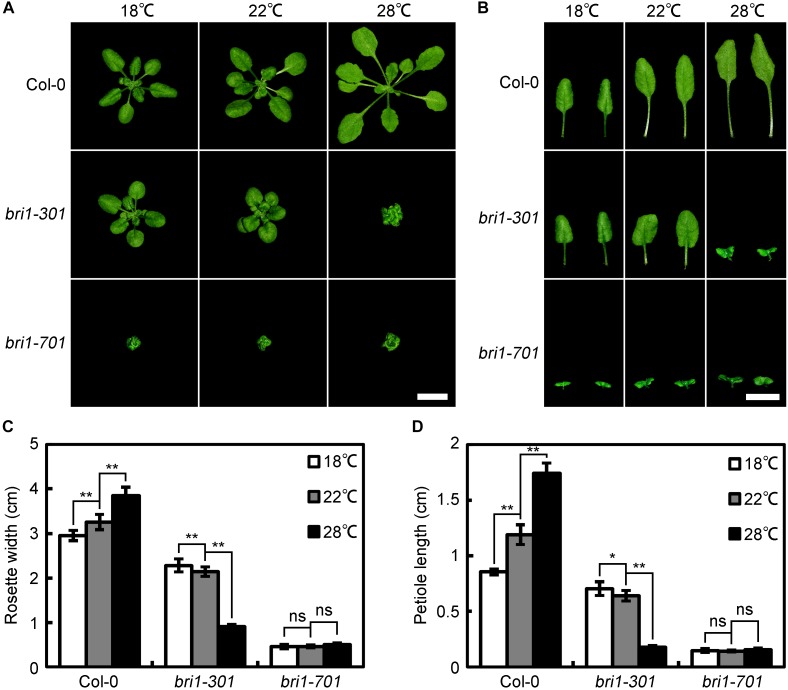
*bri1-301* growth is significantly retarded by elevated ambient temperature. **(A)** Rosette phenotypes of wild type and different *bri1* alleles grown at 18°C, 22°C, and 28°C. **(B)** Leaf phenotypes of wild type and different *bri1* alleles grown at 18°C, 22°C, and 28°C. Photographs of the rosettes and the third and fourth true leaves were taken 4 weeks after germination. Scale bars represent 1 cm. **(C)** Measurements of rosette width of plants as shown in **(A)**. **(D)** Measurements of petiole length of the third and fourth true leaves as present in **(B)**. In **(C,D)**, the data shown are means and standard deviations. The asterisks indicate statistical significance evaluated by Student’s *t*-test (^∗^*P* < 0.05, ^∗∗^*P* < 0.01), ns represents not significant. Three independent biological replicates were carried out. Similar results were obtained. One of the representative results is shown.

*bri1-301* was originally characterized as a weak *bri1* allele carrying two tandem nucleotide mutations, resulting in one amino-acid substitution, G989I (Xu et al., 2008). A previous report indicated that bri1-301 does not show any detectable activity either for autophosphorylating itself or phosphorylating its known substrate, BAK1, *in vitro* (Xu et al., 2008). The appeared weak phenotype of *bri1-301* is contradictory with other known *BRI1* kinase-dead mutants, which always show extreme morphological defects. Therefore, we are interested in studying whether the kinase activity of BRI1 is essential to the BR signaling pathway using *bri1-301* as a studying material. During our investigation, however, we unintentionally observed that the phenotypic defects of soil-grown *bri1-301* mutants largely rely on their growing temperatures. In a relatively low temperature growth chamber (18°C), *bri1-301* only shows a subtle defective phenotype compared to wild type (Figure 1). In a growth chamber normally used for Arabidopsis growth (22°C), *bri1-301* displays a weak *bri1* defective phenotype, similar to several other well-characterized *bri1* mutants such as *bri1-5, bri1-120*, and *bri1-702* (Noguchi et al., 1999; Shang et al., 2011; Sun et al., 2017) (Figure 1). Surprisingly, in a warmer growth chamber (28°C), *bri1-301* exhibits a severe defective phenotype, similar to null *bri1* mutants, such as *bri1-701*, *bri1-4*, and *bri1-709* (Noguchi et al., 1999; Gou et al., 2012; Sun et al., 2017) (Figure 1).

We also examined the phenotypes of seedlings grown on half strength of Murashige and Skoog (1/2 MS) media at three different temperatures, 18°C, 22°C, and 28°C. Under a long-day photoperiod condition (16 h light/8 h dark), warmer temperature inhibits the elongation of cotyledon petioles of *bri1-301*, resulting in compact and curled cotyledons (Supplementary Figure S1A). In darkness, hypocotyl elongation of Col-0 is stimulated by warmer temperature (Supplementary Figures S1B,C). Although *bri1-301* shows more elongated hypocotyls at 22°C, further raising temperature to 28°C can significantly inhibit the hypocotyl elongation (Supplementary Figures S1B,C).

### Warmer Temperature-Inhibited Growth of *bri1-301* Is Caused by G989I Substitution

**FIGURE 2:**
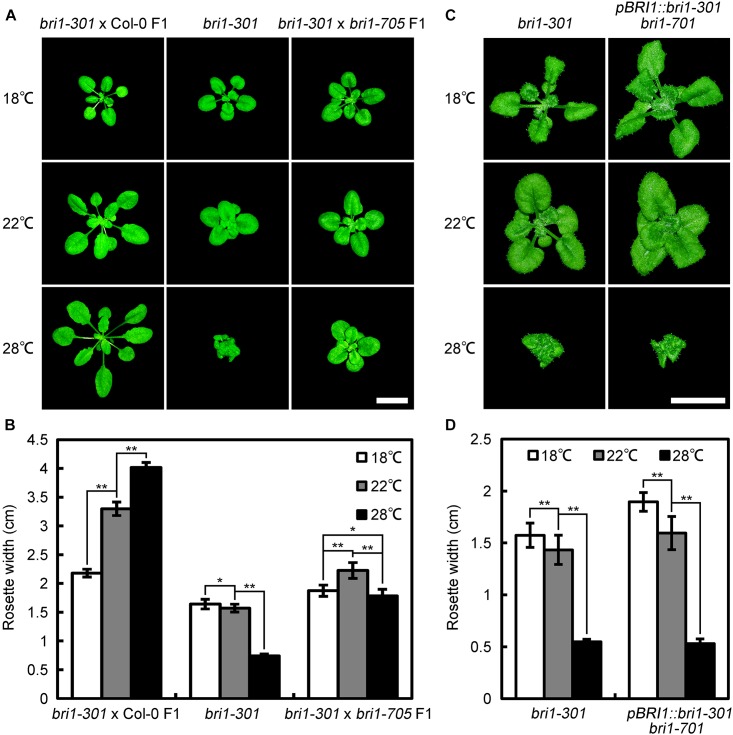
Genetic analyses demonstrated that the point mutation shown in *bri1-301* is responsible for retarded growth phenotype at elevated temperature. **(A)** Phenotypes of 4-week-old *bri1-301* and F1 hybrids grown at 18°C, 22°C, and 28°C. Scale bar represents 1 cm. **(B)** Measurements of rosette width of the plants as shown in **(A)**. **(C)** Phenotypes of 5-week-old *bri1-301* and *pBRI1:bri1-301 bri1-701* grown at 18°C, 22°C, and 28°C. Scale bar represents 1 cm. **(D)** Measurements of rosette width of the plants as shown in (A). In **(B,D)**, the data shown are means and standard deviations. The asterisks indicate statistical significance evaluated by Student’s *t*-test (^∗^*P* < 0.05, ^∗∗^*P* < 0.01). Three biological replicates were carried out. Similar results were obtained. One of the representative results is shown.

Previous studies indicated that some Arabidopsis mutants, including *zed1-D* (*hopz-eti-deficient 1-dominant*) (Wang et al., 2017), *eta1* (*enhancer of tir1-1 auxin resistance 1*) (Quint et al., 2005), *scd1-1* (*stomatal cytokinesis-defective 1-1*) (Falbel et al., 2003), exhibit growth retardation under a comparatively warmer condition. Hence, it is possible that another point mutation other than G989I in *bri1-301* is responsible for the warm-enhanced phenotype. To examine this possibility, we crossed *bri1-301* with Col-0 and a newly identified subtle *bri1* mutant, *bri1-705*, and analyzed the phenotypes of F1 individuals at 18°C, 22°C, and 28°C, respectively (Sun et al., 2017). We found that *bri1-301* × Col-0 F1 heterozygous individuals show fully complemented phenotype similar to wild type, Col-0, whereas *bri1-301* ×*bri1-705* F1 individuals show a slightly retarded growing phenotype at 28°C (Figures 1A,C, 2A,B). We also generated transgenic plants harboring *bri1-301* coding sequence driven by a *BRI1* native promoter in a *bri1-701* background. As expected, the transgenic lines recapitulated a temperature-dependent phenotype as *bri1-301* (Figures 2C,D). These observations suggest that the warmer temperature-inhibited growth of *bri1-301* is indeed caused by the G989I substitution within BRI1 but unlikely by other unknown mutations within the *bri1-301* genome.

### Temperature-Dependent *bri1-301* Severity Is Allele Specific

**FIGURE 3:**
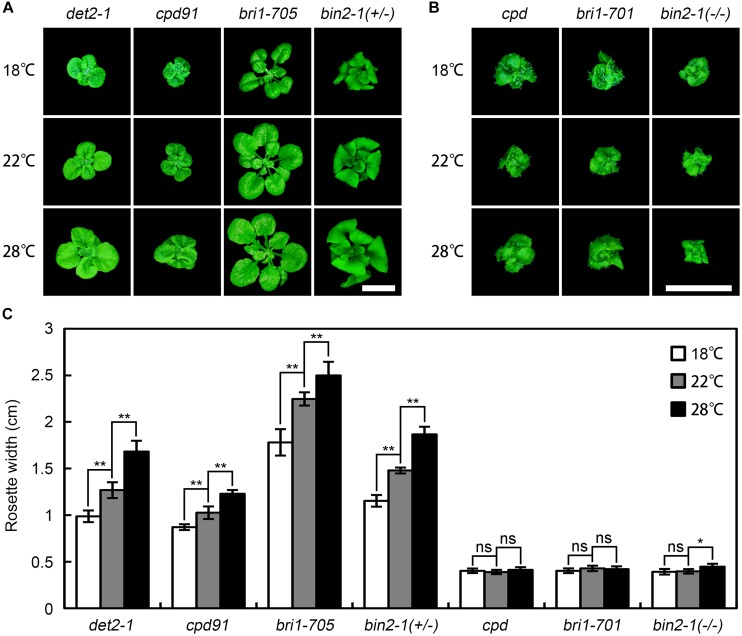
Effects of elevated ambient temperature on growth of other BR mutants. **(A,B)** Phenotypes of 4-week-old BR mutants grown at 18°C, 22°C, and 28°C. Scale bars represent 1 cm. **(C)** Measurements of rosette width of the plants as shown in **(A,B)**. The data shown are means and standard deviations. The asterisks indicate statistical significance evaluated by Student’s *t*-test (^∗^*P* < 0.05, ^∗∗^*P* < 0.01), ns represents not significant. Three independent biological replicates were carried out. Similar results were obtained. Only one of the representative results is shown.

To test whether the temperature-dependent *bri1-301* severity is allele specific, we examined the phenotypes of a number of BR deficient and signaling mutants at 18°C, 22°C, and 28°C. We found that warmer temperature generally promotes rosette growth of all other weak BR mutants tested at varying degrees, but it appears no obvious effect on the growth of null *bri1* alleles (Figure 3). We also mutated the G989 residue of BRI1 to other amino acids through site-directed mutagenesis and introduced the mutated *bri1* coding sequences driven by the *BRI1* native promoter into *bri1-701*. We successfully generated 11 G989-C, D, E, F, H, I, L, M, Q, S, or Y transgenic plants. To our surprise, all of the G989- C, D, E, F, H, L, M, Q, S, or Y transgenic plants failed to mimic the warm-inhibited growth phenotype of *bri1-301* except for the G989I transgenic plants (Supplementary Figure S2). These results suggest that warm-enhanced growth defects of *bri1-301* is both allele and amino acid specific.

### *bri1-301* Is Sensitive to the BL Treatment at Lower Temperature but Not at Warmer Temperature

**FIGURE 4:**
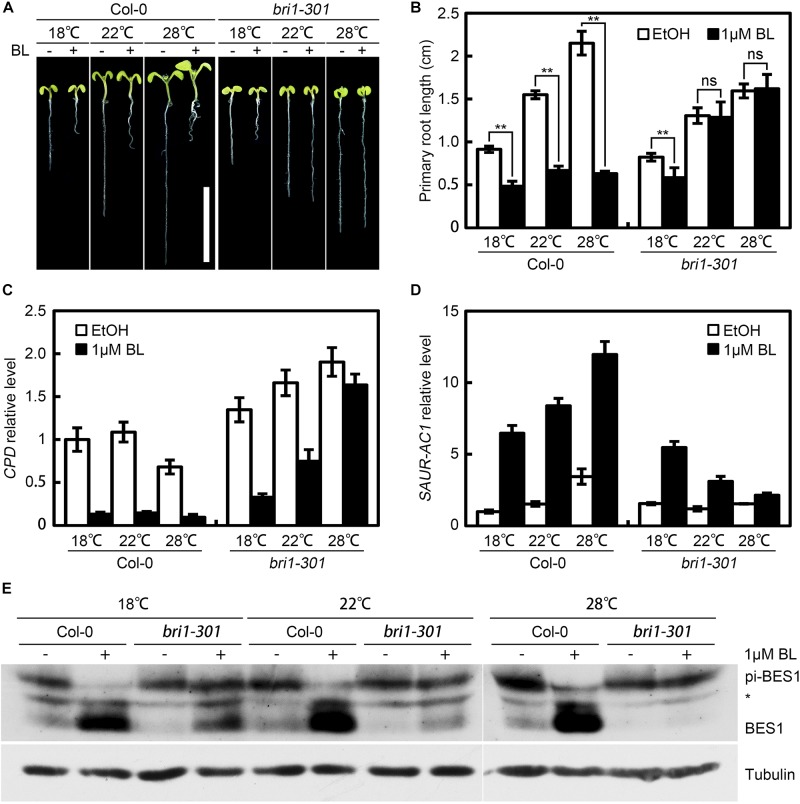
*bri1-301* is sensitive to BL at lower temperature but insensitive to BL at higher temperature. **(A)** Root growth inhibition assay of Col-0 and *bri1-301* at 18°C, 22°C, and 28°C. Seven-day-old seedlings grown on 1/2 MS media supplemented with or without 1 μM 24-epiBL were photographed. Scale bar represents 1 cm. **(B)** Measurements of primary root length of the seedlings as shown in **(A)**. The asterisks indicate statistical significance evaluated by Student’s *t*-test (^∗∗^*P* < 0.01), ns represents not significant. Three independent biological replicates were carried out. Similar results were obtained. One of the representative results is shown. **(C,D)** Transcriptional responses of *CPD* **(C)** and *SAUR-AC1* **(D)** to 24-epiBL treatment at three different temperatures. *ACTIN2* was used as an internal control. Three independent biological replicates were carried out. Similar results were obtained. One of the representative results is shown. **(E)** BES1 phosphorylation analysis in response to exogenous 24-epiBL at different temperatures. The upper and lower bands represent phosphorylated and dephosphorylated BES1, respectively. The middle bands marked by an asterisk indicate non-specific signals generated by anti-BES1 serum. Tubulin was used as the loading control.

The phenotypic resemblance of 28°C-grown *bri1-301* to *bri1-701* suggests that BR signal transduction pathway in *bri1-301* is disrupted at 28°C. To test this hypothesis, we compared the BR responses of Col-0 and *bri1-301* under various temperature conditions. Root growth inhibition assay indicated that when grown at 18°C (Clouse et al., 1996), the primary root growth of *bri1-301* is inhibited by exogenously applied BL, the most active form of BRs (Figures 4A,B). However, such inhibitory effect was not observed at 22°C and 28°C (Figures 4A,B). Consistently, quantitative RT-PCR assay showed that the expression levels of *CPD* and *SAUR-AC1* are significantly increased and decreased, respectively, in *bri1-301* compared to those in wild type at 28°C (Supplementary Figure S3). Meanwhile, when raising temperature, the feedback regulations of *CPD* and *SAUR-AC1* by exogenously applied BL were gradually attenuated (Goda et al., 2002) (Figures 4C,D). Western blot analyses revealed that the BL induced accumulation of unphosphorylated BES1 in *bri1-301* can be detected at 18°C and 22°C but not at 28°C (Yin et al., 2002) (Figure 4E). In summary, these results demonstrated that warmer temperature at 28°C inhibits BR signaling in *bri1-301*.

### At Lower Temperature but Not at Warmer Temperature, BAK1 Can Promote the Growth of *bri1-301*

**FIGURE 5:**
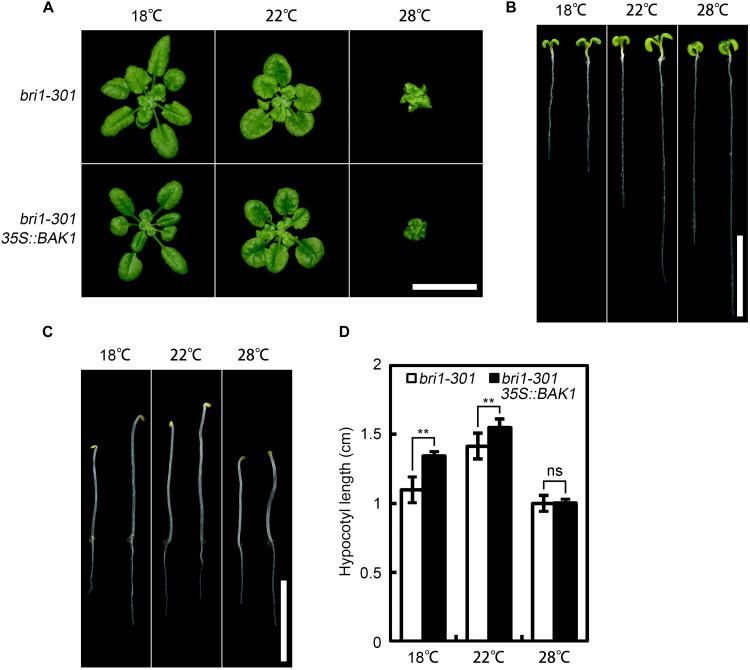
Overexpression of *BAK1* can suppress *bri1-301* at lower temperature but not at higher temperature. **(A)** Phenotypes of 4-week-old *bri1-301* and *35S:BAK1 bri1-301* plants grown at 18°C, 22°C, and 28°C. **(B)** Phenotypes of 7-day-old light-grown seedlings on 1/2 MS plates at 18°C, 22°C, and 28°C. **(C)** Phenotypes of 4-day-old dark-grown seedlings on 1/2 MS plates at 18°C, 22°C, and 28°C. For each temperature panel shown in **(B,C)**, the left seedling represents a *bri1-301* and the right one represents a *35S:BAK1 bri1-301* transgenic plant. Scale bars represent 1 cm. **(D)** Measurements of hypocotyl length of the seedlings as shown in **(C)**. The data shown are means and standard deviations. The asterisks indicate statistical significance evaluated by Student’s *t*-test (^∗∗^*P* < 0.01), ns represents not significant.

As co-receptors, BAK1 and its homologs play indispensable roles in the initiation of BR signal transduction (Gou et al., 2012). Overexpression of *BAK1* can suppress the defective phenotype of weak but not null *bri1* alleles (Li et al., 2002), suggesting the requirement of at least a partially functional BRI1 for the function of BAK1. To test whether bri1-301 is functional at warmer temperature, we overexpressed *BAK1* in *bri1-301* and analyzed the phenotypes of the transgenic plants at three aforementioned different temperatures. Our results indicated that overexpression of *BAK1* can partially suppress the defective phenotype of *bri1-301* at both 18°C and 22°C but not at 28°C (Figure 5). These genetic results confirmed that bri1-301 lost most of its biological function at 28°C.

### BR Signaling Initiation in *bri1-301* Is Impaired Under a Warmer Temperature Condition

**FIGURE 6:**
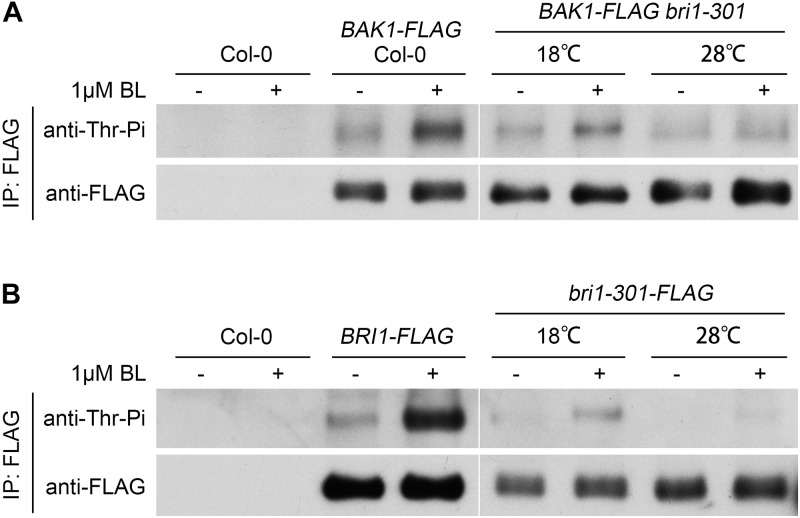
Exogenous application of 24-epiBL can induce the phosphorylation levels of both BAK1 and bri1-301 at 18°C, but at 28°C such effect is significantly reduced. **(A)** Western blotting analyses of BL-induced phosphorylation of BAK1 in Col-0 and *bri1-301* at two indicated temperatures. **(B)** Western blotting analyses of BL-induced phosphorylation of BRI1 and bri1-301 at two indicated temperatures. Transgenic seedlings were treated with or without 1 μM 24-epiBL for 1.5 h at the same temperature conditions they grown.

Successful BR signal initiation depends on the activation of the BR receptor BRI1 and co-receptor BAK1, which can be analyzed by their phosphorylation status after the treatment of the ligand, BL. We tested the phosphorylation levels of BRI1, bri1-301, and BAK1 in response to exogenous applied BL at different temperatures. The phosphorylation levels of BRI1 and BAK1 from wild type can be significantly induced by the BL treatment (Figure 6), as previously reported (Wang et al., 2005a). The phosphorylation levels of bri1-301 and BAK1 from *bri1-301* can be induced by BL at 18°C. The degree of induction, however, is greatly reduced compared to their corresponding proteins from wild type. The induction of phosphorylation status of bri1-301 and BAK1 in *bri1-301* is almost invisible at 28°C (Figure 6). These results further demonstrated that warmer temperature blocks the BR signaling initiation in *bri1-301*.

### bri1-301 Is Less Abundant Than BRI1 and Its Stability Is Greatly Reduced at 28°C

**FIGURE 7:**
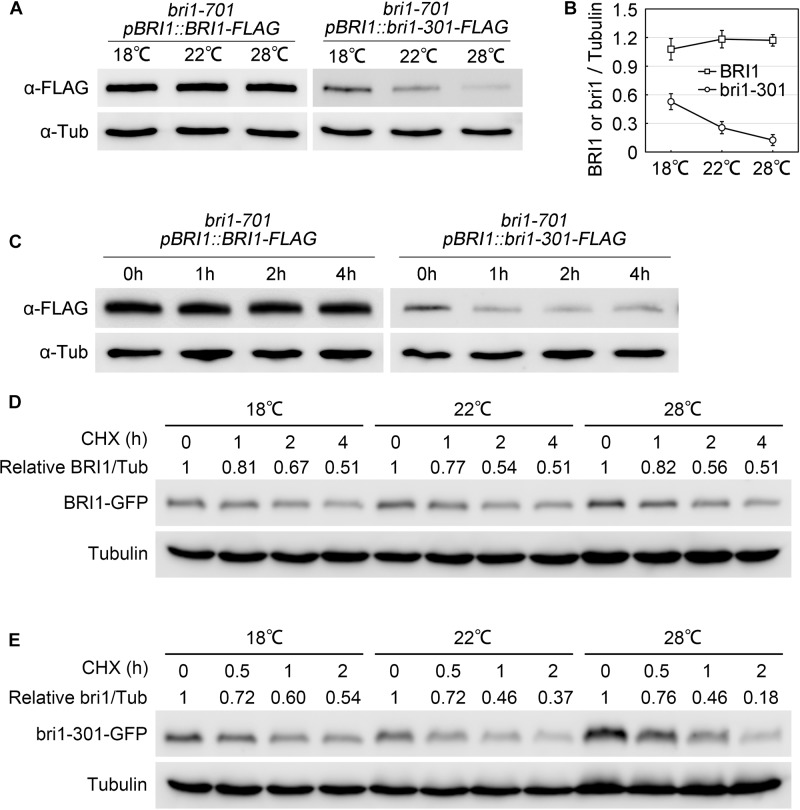
Stability of bri1-301 protein is negatively regulated by elevated temperature. **(A)** Western blot analyses of total protein extracts from aerial parts of 3-week-old *pBRI1::BRI1-FLAG bri1-701* and *pBRI1::bri1-301-FLAG bri1-701* transgenic plants. Tubulin was used as the loading control. **(B)** Relative BRI1 or bri1-301 protein levels were calculated as the average ratios (±SD) of Bri1/Tubulin signals in **(A)** from three independent experiments. **(C)** BRI1 and bri1-301 protein accumulation under short-term warm treatment. Three-week-old 18°C-grown *pBRI1::BRI1-FLAG bri1-701* and *pBRI1::bri1-301-FLAG bri1-701* transgenic plants were transferred into a 28°C growth chamber and the aerial parts were harvested at the indicated time points. Tubulin was used as the loading control. Three independent biological replicates were carried out. Similar results were obtained. One of the representative results is shown. **(D,E)** Abundance of BRI1 **(D)** and bri1-301 **(E)** in response to a time-course CHX treatment at three temperatures. Seven-day-old *pBRI1::BRI1-GFP bri1-701* and *pBRI1::bri1-301-GFP bri1-701* transgenic seedlings were used for the CHX treatment, total protein extracts were used for Western blot analyses. Tubulin was used as the loading control. Relative BRI1 or bri1-301 protein levels were calculated as ratios of BRI1/Tubulin.

**FIGURE 8:**
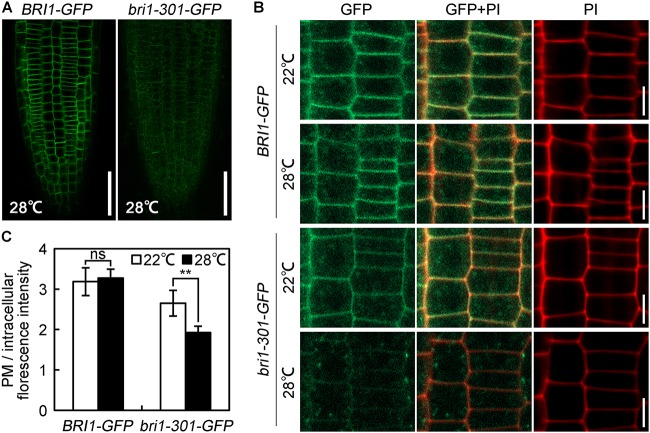
Warm-enhanced endocytosis of bri1-301 protein. **(A)** Confocal microscopic images of 28°C-grown seedlings. Roots were immediately scanned by confocal microscope after mounting. Scale bars represent 50 μm. **(B)** The subcellular localization of BRI1 and bri1-301 protein. Roots were stained with PI and then were immediately scanned by confocal microscope. Scale bar represents 10 μm. **(C)** Quantification of the ratio between the PM and the intracellular BRI1/bri1-301-GFP signal intensities from images shown in **(B)**. The data shown are means and standard deviations. The asterisks indicate statistical significance evaluated by Student’s *t*-test (^∗∗^*P* < 0.01), ns represents not significant. At least 10 roots for each sample were analyzed. Similar results were obtained. One of the representative picture for each treatment is shown.

To elucidate the molecular mechanisms regulating bri1-301 functionality at various temperatures, we first evaluated the expression levels of *BRI1* and *bri1-301* and their responses to elevated temperatures. *BRI1* and *bri1-301* showed similar expression levels at 18°C and 22°C. At 28°C, *bri1-301* is expressed even more than *BRI1* (Supplementary Figure S4A). These results indicated that the severe phenotype of *bri1-301* at 28°C is not caused by reducing transcription levels of *bri1-301*. The regulation is therefore likely at a post-transcriptional level. To test whether the protein abundance of the BR receptor has been altered in *bri1-301* compared to wild type, we carried out a semi-quantitative Western blot analysis to detect the BR receptor proteins in wild type and *bri1-301* by using an anti-BRI1 antibody. We found that the abundance of bri1-301 is much lower than BRI1, suggesting a reduced stability of bri1-301 than BRI1 (Supplementary Figure S4B). Due to low signal intensity of bri1-301 as shown in a Western blot analysis using an anti-BRI1 antibody (Supplementary Figure S4B), we therefore analyzed the impact of different temperatures on FLAG-tagged versions of BRI1 and bri1-301 proteins by using an anti-FLAG antibody. Our data showed that wild-type BRI1 protein level does not respond to elevated ambient temperature, whereas bri1-301 protein abundance is dramatically reduced at 28°C relative to those at 18°C or 22°C (Figures 7A,B and Supplementary Figure S4C). Transferring *bri1-701 pBRI1::bri1-301-FLAG* from 18 to 28°C for only 1 h can significantly reduce the abundance of bri1-301-FLAG (Figure 7C), suggesting a negative role of warmer temperature on bri1-301 protein stability. To further confirm this hypothesis, we evaluated BRI1/bri1-301-GFP protein abundance in *BRI1-GFP* and *bri1-301-GFP* transgenic seedlings treated with cycloheximide (CHX), a protein biosynthesis inhibitor, at three different temperatures. Our results showed that warmer temperature does not affect BRI1 stability. BRI1 abundance drops in similar patterns at different temperatures when protein biosynthesis is blocked (Figure 7D). In comparison, bri1-301 abundance drops much quicker under warmer temperature conditions (Figure 7E). Moreover, confocal microscopic analysis showed that the bri1-301 protein was accumulated more in the endosomal compartments compared to BRI1 when exposed to a warmer condition (Figure 8). In conclusion, bri1-301 appeared less stable than BRI1 and warmer temperature could further destabilize bri1-301.

## Discussion

### Kinase Activity of bri1-301 Is Crucial for Its Biological Function

As a receptor kinase, BRI1 kinase activity is essential to the initiation of successful BR signaling (Friedrichsen et al., 2000; Wang et al., 2008; Oh et al., 2009; Jaillais et al., 2011). The crucial role of kinase activity for BRI1 function has been demonstrated by severe morphological defects of null *bri1* mutant alleles, such as *bri1-1*, *bri1-101*, *bri1-117*, and *bri1-703* (Clouse et al., 1996; Li and Chory, 1997; Friedrichsen et al., 2000; Sun et al., 2017). Consistently, mutants with partial BRI1 kinase activities, such as *bri1-702*, usually show weak defective phenotypes (Sun et al., 2017). Interestingly, *in vitro* kinase autophosphorylation assay indicated that bri1-8/108 and bri1-301 proteins do not show any kinase activities, but *bri1-8/108* and *bri1-301* plants exhibit even weaker defective phenotypes than *bri1-702* (Noguchi et al., 1999; Xu et al., 2008; Sun et al., 2017). Because of these exceptions, there was an argument debating whether the kinase activity of BRI1 is truly essential to plant growth and development (Xu et al., 2008). In one of our previous reports, we showed that phosphorylation level of BAK1 in *bri1-301* is much higher than that in *bri1-701*, a null T-DNA insertional mutant of *BRI1* (Sun et al., 2017). In addition, the phosphorylation level of BAK1 in *bri1-301* can be significantly induced by exogenously applied BL. These results indirectly suggested bri1-301 is a partially functional receptor *in vivo* (Sun et al., 2017). Here, we provide additional evidence to show that, under normal Arabidopsis laboratory growth conditions (22°C, 16 h light/8 h dark), *bri1-301* mutant is able to respond to exogenously applied BL, indicated by ligand-induced both target gene feedback regulation and dephosphorylated BES1 accumulation analyses (Figures 4C–E). *In vivo* phosphorylation analysis showed that the weak phosphorylation level of bri1-301 can be detected and elevated by exogenous BL treatment (Figure 6). In addition, although the mRNA levels of *bri1-301* and *BRI1* are equivalent in *bri1-301* and wild type (Supplementary Figure S4A), the bri1-301 protein abundance in *bri1-301* is significantly reduced compared to BRI1 in wild type (Figures 7A,B and Supplementary Figure S4B), suggesting that bri1-301 is a highly unstable protein. These data imply that the reduced protein abundance and partially impaired kinase activity likely have caused the weak phenotype of *bri1-301*. Our analyses clarified the early question regarding the importance of BRI1 kinase activity (Xu et al., 2008).

### Warmer Condition Destabilizes bri1-301 Receptor, Leading to Severer Defective Phenotype of *bri1-301*

During our phenotypic analyses of *bri1-301*, we accidentally identified that the morphological defects of *bri1-301* is temperature-dependent. When grown at lower temperature (18°C), *bri1-301* shows a subtle defective phenotype. Although the BRI1 protein level in *bri1-301* is reduced to approximately 50% that of wild type, such amount of bri1-301 seems sufficient to maintain normal growth and development (Figures 1, 7A,B). When grown at warmer temperature (28°C), however, *bri1-301* shows a severer defective phenotype similar to that of a strong *bri1* allele, such as *bri1-701* (Figure 1). The bri1-301 abundance is further reduced to about 10% the level of BRI1 in wild type (Figure 7). In addition, warmer temperature also increases internalization of bri1-301 (Figures 8B,C). Therefore, these results suggest that warm-temperature-induced bri1-301 protein internalization and degradation is likely the cause of the severer phenotype of *bri1-301*.

### The bri1-301 Protein Partially Undergoes the ERQC Process

BRI1 was reported to undergo a highly conserved endoplasmic reticulum-mediated protein quality control (ERQC) mechanism before further trafficking to plasma membrane. Some missense BRI1 receptors with mutations in the extracellular domain, such as bri1-5 and bri1-9, are ER-retained and are subjected to ER-associated degradation (ERAD), resulting in low protein abundance (Liu and Li, 2014). Although bri1-301 also shows a decreased stability and low protein abundance, the quality control mechanisms of bri1-301 seem to be different from those of bri1-5 and bri1-9. Firstly, the amino acid substitution in bri1-301 occurs in the cytoplasmic domain, whereas the mutations in bri1-5 and bri1-9 are in the extracellular domain. The extracellular domain of BRI1 is toward to the lumen side of ER during trafficking. Secondly, bri1-301 is partially retained by glycan-dependent mechanism. Our results showed that bri1-301 displays a partial cleavage pattern when treated with endoglycosidase H (Endo H) (Supplementary Figure S5), an enzyme can remove high-mannose-type glycans of ER-localized glycoproteins but cannot cleave Golgi-processed glycans (Maley et al., 1989). The Endo H susceptibility of bri1-301 is much lower than bri1-5 and bri1-9, two well characterized Endo H substrates (Hong et al., 2009; Jin et al., 2009; Liu and Li, 2014). Thirdly, previous studies indicated that inactivating ERQC by disrupting EBS1/UGGT can suppress the defective phenotype of *bri1-9* but not *bri1-301* (Jin et al., 2007). In addition, our confocal analysis indicated that at 22°C, bri1-301-GFP, like BRI1-GFP, is mainly localized on the plasma membrane instead of retaining in ER (Figures 8B,C). Our observation suggests that the instability of bri1-301 is likely controlled by multiple mechanisms including the glycan-dependent ERQC.

### Thermal-Enhanced bri1-301 Instability Suggests a Plasma Membrane Quality Control System in Plants

According to studies in yeast and mammalian cells, non-native membrane proteins are normally eliminated by ERQC systems. By contrast, the non-native membrane proteins escaped from the ERQC are preferentially eliminated by plasma membrane quality control (PMQC) mechanisms (Okiyoneda et al., 2011; Apaja and Lukacs, 2014). Many mutant variants of membrane proteins such as PM H^+^-ATPase (Pma1), α factor receptor (Ste2-3), and cystic fibrosis transmembrane conductance regulator (Δ508CFTR) are rapidly internalized and degraded from cell surface in a temperature-sensitive manner (Chang and Fink, 1995; Jenness et al., 1997; Sharma et al., 2001). The destabilizing point mutations in these membrane proteins are predominantly localized in the cytoplasmic and transmembrane segments (Okiyoneda et al., 2011). Our analysis indicated that bri1-301 can be partially recognized by ERQC machinery. As a result, a great deal of bri1-301 can escape from the ER and reach its destination, the plasma membrane under a normal Arabidopsis growing condition. Some similarities can be found between bri1-301 and well-studied animal PMQC proteins: (1) they share cytoplasmic mutations; (2) these mutations can decrease protein stability; and (3) they have thermal-enhanced mutant phenotype, protein internalization and degradation properties. Based on these findings, we propose the existence of PMQC system for BRI1 and possibly other RLKs in plants.

### *bri1-301* Can Serve as a Model to Study PMQC Mechanism in Plants

Although a number of PMQC proteins have been found in yeast and mammalian cells, it remains unclear how unfolded membrane proteins are recognized and removed from the plasma membrane (Okiyoneda et al., 2011). Recent studies proposed a general PMQC mechanism for removing the conformationally unstable proteins from the PM via ubiquitination, endocytosis, and lysosomal degradation (Okiyoneda et al., 2011; Apaja and Lukacs, 2014). In plants, PM-localized BRI1 has been reported to be ubiquitinated, internalized, and sorted to recycling or degradation machinery (Russinova et al., 2004; Geldner et al., 2007; Martins et al., 2015; Zhou et al., 2018). These processes implicate two plant U-box E3 ubiquitin ligases, PUB12 and PUB13 (Zhou et al., 2018); several components involved in endocytosis, AP-2, TPLATE, and GNOM (Irani et al., 2012; Di Rubbo et al., 2013; Gadeyne et al., 2014); and ALIX1, a cytoplasmic protein participating in vacuolar sorting (Cardona-Lopez et al., 2015). Therefore, it is intriguing to investigate the functional relevance between BRI1 membrane trafficking and its conformational change during BR perception and signaling initiation. As an unstable BR receptor, especially at warmer temperature, bri1-301 can be used as a model to study PMQC and its function in maintaining BRI1 integrity.

## Author Contributions

JL conceived the research plans, designed the experiments, and edited the manuscript. MLv, MLi, WC, YW, CS and HY performed the experiments. MLv analyzed the data and prepared the manuscript draft. KH provided comments for the manuscript.

## Conflict of Interest Statement

The authors declare that the research was conducted in the absence of any commercial or financial relationships that could be construed as a potential conflict of interest.

## Footnotes

1 http://rsb.info.nih.gov/ij/
