# Supplementary material for: Thermal-Enhanced bri1-301 Instability Reveals a Plasma Membrane Protein Quality Control System in Plants
Source: Front Plant Sci. 2018 Nov 6;9:1620. doi: 10.3389/fpls.2018.01620 (PMC6232910; doi:10.3389/fpls.2018.01620)
Supplement: Supplementary file 1 [file Table_1.DOCX]

**Table S1. Primers used in this research.**

| Used for Genotyping. | |
| --- | --- |
| bri1-301-CAPS-F | AAATCTTGTGCCTCTTCTTGGT |
| bri1-301-CAPS-R | GGGTCAAACACATCGCTAATC |
| bri1-701-GT-F | GATTCCTTTCCTCGGAGATTG |
| bri1-701-GT-R | TTGTTCGGATCTGATTCCTTG |
| LBb1.3 | ATTTTGCCGATTTCGGAAC |
| BRI1-7R | ATCTATTCTCACTTTCCCATTCC |
| Used for quantitative RT-PCR. | |
| ACT2-Q-F | TGTGCCAATCTACGAGGGTTT |
| ACT2-Q-R | TTTCCCGCTCTGCTGTTGT |
| BRI1-Q-F | CTCTCCTGTCTCTCACCGGA |
| BRI1-Q-R | GCACTTGAAGCCAGAAACGG |
| CPD-Q-F | GAGACGCTACGAGTGGCTAA |
| CPD-Q-R | GCATCTTTGAAGTGGTTTGGG |
| SAUR_AC1-Q-F | AGATATGTGGTGCCGGTTTC |
| SAUR_AC1-Q-R | TTGTTAAGCCGCCCATTG |
| Used for gene cloning. | |
| attB1 | GGGGACAAGTTTGTACAAAAAAGCAGGCTTC |
| attB2 | GGGGACCACTTTGTACAAGAAAGCTGGGTC |
| BRI1/bri1-nsc-F | AAAAAGCAGGCTTCATGAAGACTTTTTCAAGCTTCTTT |
| BRI1/bri1-nsc-R | AGAAAGCTGGGTCTAATTTTCCTTCAGGAACTTCTTT |
| G989A-F | CGGAAGATTGCGATAGCATCAGCTAGAGGGCT |
| G989A-R | AGCCCTCTAGCTGATGCTATCGCAATCTTCCG |
| G989C-F | CGGAAGATTGCGATATGTTCAGCTAGAGGGCT |
| G989C-R | AGCCCTCTAGCTGAACATATCGCAATCTTCCG |
| G989D-F | CGGAAGATTGCGATAGATTCAGCTAGAGGGCT |
| G989D-R | AGCCCTCTAGCTGAATCTATCGCAATCTTCCG |
| G989E-F | CGGAAGATTGCGATAGAATCAGCTAGAGGGCT |
| G989E-R | AGCCCTCTAGCTGATTCTATCGCAATCTTCCG |
| G989F-F | CGGAAGATTGCGATATTTTCAGCTAGAGGGCT |
| G989F-R | AGCCCTCTAGCTGAAAATATCGCAATCTTCCG |
| G989H-F | CGGAAGATTGCGATACATTCAGCTAGAGGGCT |
| G989H-R | AGCCCTCTAGCTGAATGTATCGCAATCTTCCG |
| G989I-F | CGGAAGATTGCGATAATATCAGCTAGAGGGCT |
| G989I-R | AGCCCTCTAGCTGATATTATCGCAATCTTCCG |
| G989K-F | CGGAAGATTGCGATAAAATCAGCTAGAGGGCT |
| G989K-R | AGCCCTCTAGCTGATTTTATCGCAATCTTCCG |
| G989L-F | CGGAAGATTGCGATACTATCAGCTAGAGGGCT |
| G989L-R | AGCCCTCTAGCTGATAGTATCGCAATCTTCCG |
| G989M-F | CGGAAGATTGCGATAATGTCAGCTAGAGGGCT |
| G989M-R | AGCCCTCTAGCTGACATTATCGCAATCTTCCG |
| G989N-F | CGGAAGATTGCGATAAATTCAGCTAGAGGGCT |
| G989N-R | AGCCCTCTAGCTGAATTTATCGCAATCTTCCG |
| G989P-F | CGGAAGATTGCGATACCATCAGCTAGAGGGCT |
| G989P-R | AGCCCTCTAGCTGATGGTATCGCAATCTTCCG |
| G989Q-F | CGGAAGATTGCGATACAATCAGCTAGAGGGCT |
| G989Q-R | AGCCCTCTAGCTGATTGTATCGCAATCTTCCG |
| G989R-F | CGGAAGATTGCGATACGATCAGCTAGAGGGCT |
| G989R-R | AGCCCTCTAGCTGATCGTATCGCAATCTTCCG |
| G989S-F | CGGAAGATTGCGATAAGTTCAGCTAGAGGGCT |
| G989S-R | AGCCCTCTAGCTGAACTTATCGCAATCTTCCG |
| G989T-F | CGGAAGATTGCGATAACATCAGCTAGAGGGCT |
| G989T-R | AGCCCTCTAGCTGATGTTATCGCAATCTTCCG |
| G989V-F | CGGAAGATTGCGATAGTATCAGCTAGAGGGCT |
| G989V-R | AGCCCTCTAGCTGATACTATCGCAATCTTCCG |
| G989W-F | CGGAAGATTGCGATATGGTCAGCTAGAGGGCT |
| G989W-R | AGCCCTCTAGCTGACCATATCGCAATCTTCCG |
| G989Y-F | CGGAAGATTGCGATATATTCAGCTAGAGGGCT |
| G989Y-R | AGCCCTCTAGCTGAATATATCGCAATCTTCCG |
