# Supplementary material for: Thermal-Enhanced bri1-301 Instability Reveals a Plasma Membrane Protein Quality Control System in Plants
Source: Front Plant Sci. 2018 Nov 6;9:1620. doi: 10.3389/fpls.2018.01620 (PMC6232910; doi:10.3389/fpls.2018.01620)
Supplement: Supplementary file 2 [file Data_Sheet_1.docx]

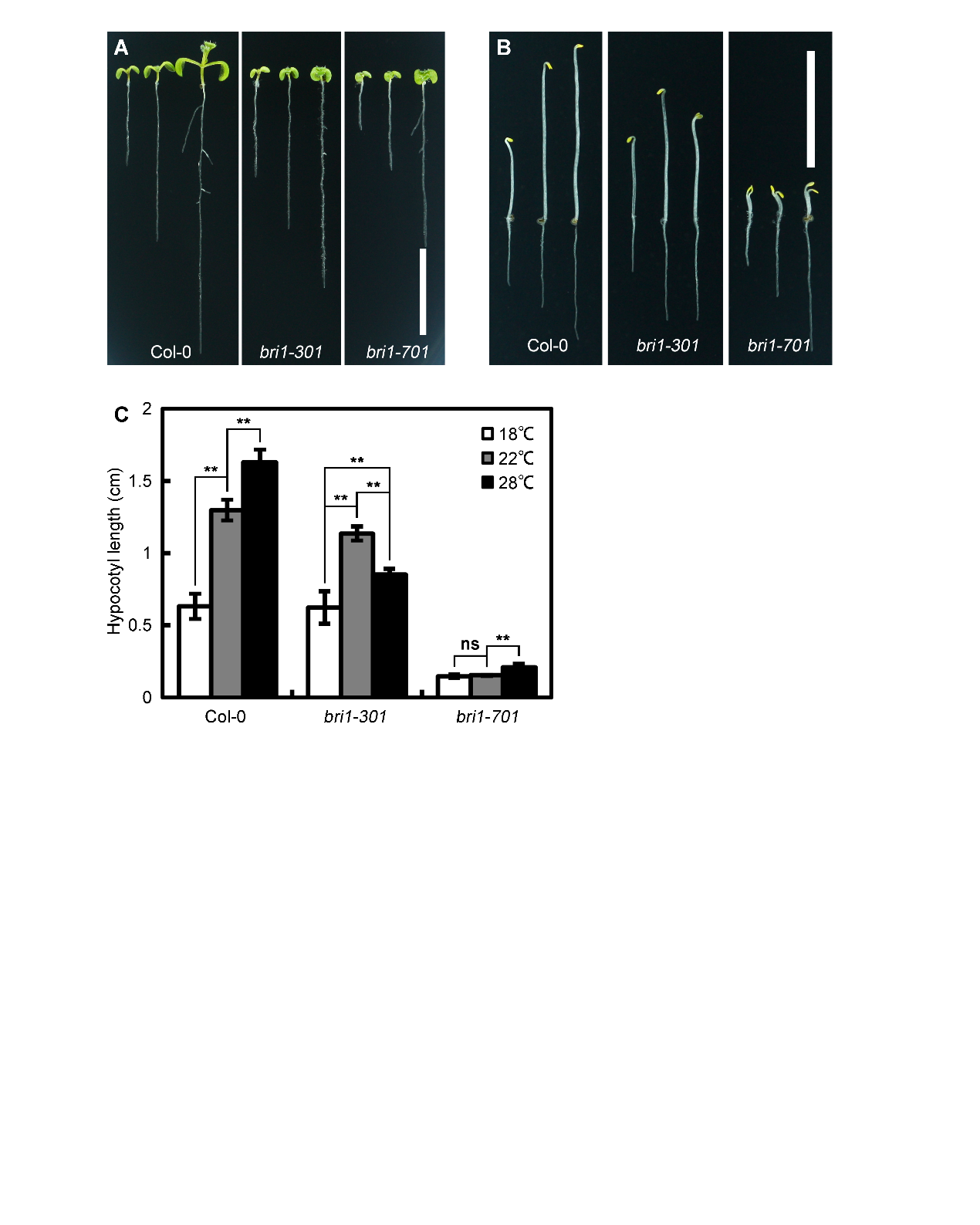


**Figure S1.** **Growth of *bri1-301* is significantly retarded by elevated ambient temperature.** **(A)** Phenotypes of 7-day-old seedlings grown on 1/2 MS plates at 18˚C, 22˚C and 28˚C. Scale bar represents 1 cm. **(B)** Phenotypes of 4-day-old de-etiolated seedlings grown on 1/2 MS plates at 18˚C, 22˚C and 28˚C under darkness. Scale bar represents 1 cm. Seedlings grown at 18˚C, 22˚C, and 28˚C are displayed from left to right in each panel from (A) and (B). **(C)** Quantitative analysis of hypocotyl length of seedlings as shown in (B). The data shown are means and standard deviations. The asterisks indicate statistical significance evaluated by Student’s *t*-test (***P* < 0.01), ns represents not significant. Three independent biological replicates were carried out. Similar results were obtained. One of the representative results is shown.


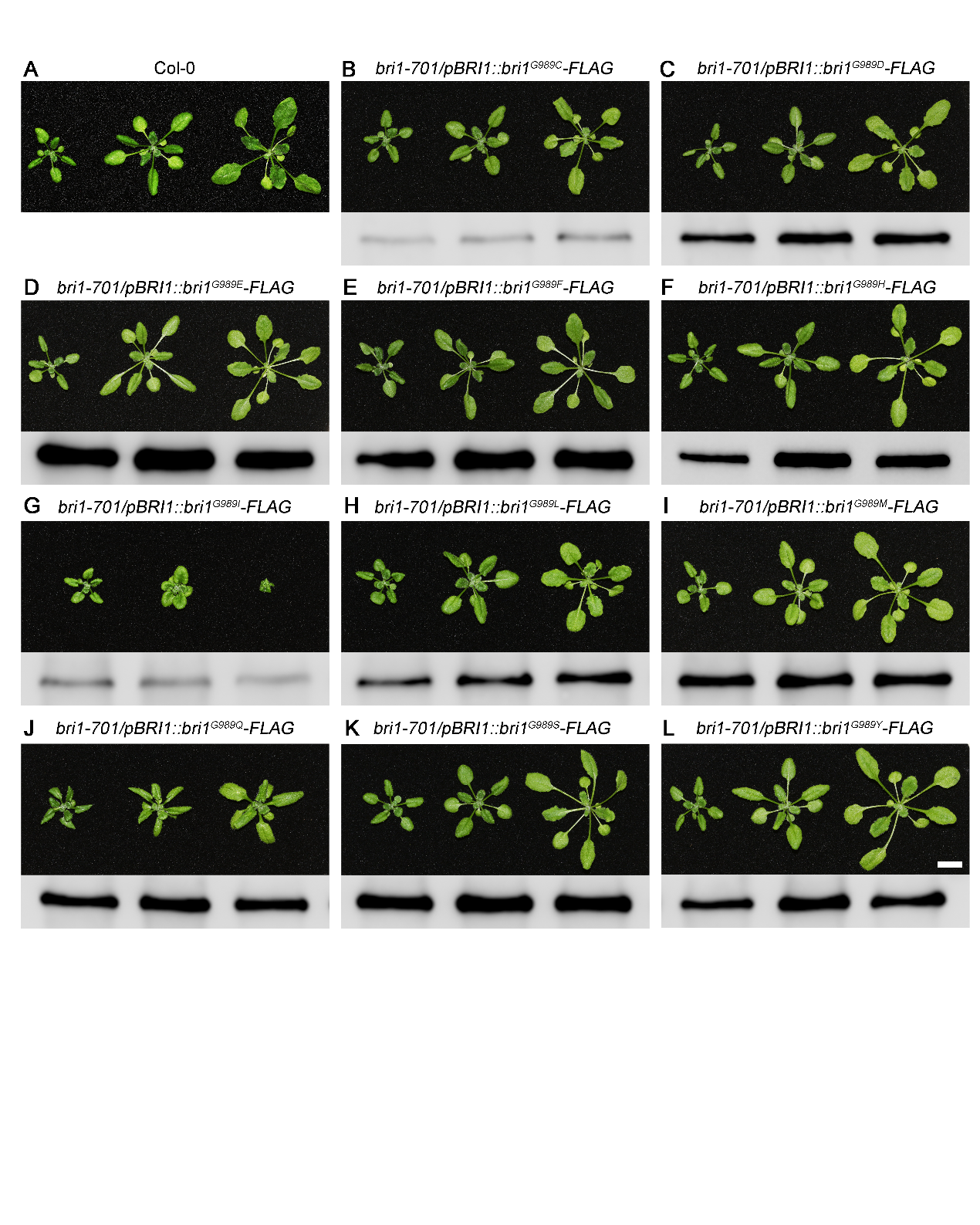


**Figure S2. Thermo-inhibition of *bri1-301* growth is amino acid specific.** The amino acid Gly 989 of BRI1 protein was artificially substituted to other amino acids including Cys **(B)**, Asp **(C)**, Asn **(D)**, Phe **(E)**, His **(F)**, Ile **(G)**, Leu **(H)**, Met **(I)**, Gln **(J)**, Ser **(K)**, and Tyr **(L)**. Then the constructs were introduced into *bri1-701*. Three-week-old plants grown at 18˚C, 22˚C, and 28˚C (from left to right in each image) were photographed.


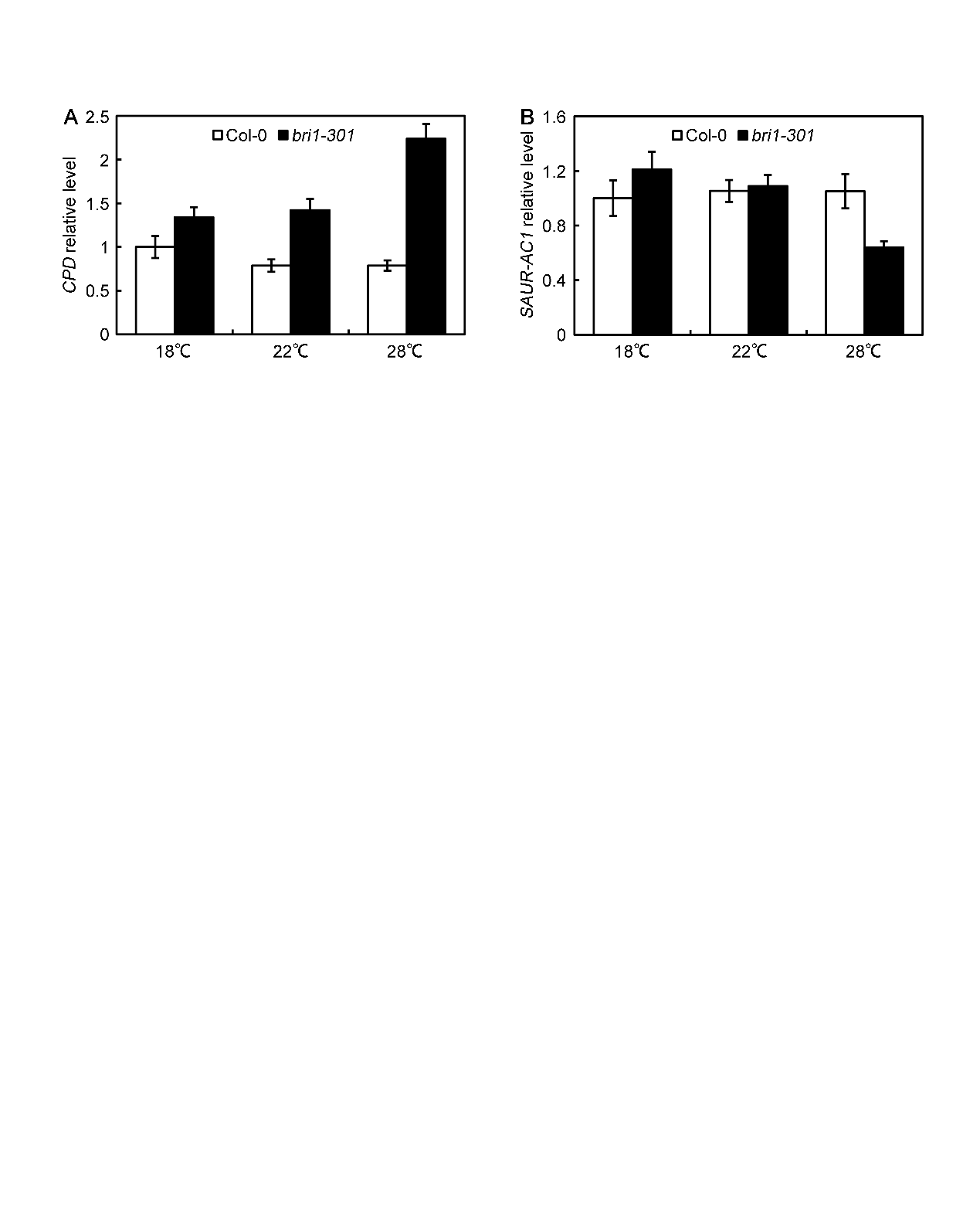


**Figure S3. BR signaling in *bri1-301* is attenuated when grown at 28˚C.** Transcription levels of *CPD* **(A)** and *SAUR-AC1* **(B)** at three different temperatures. *ACTIN2* was used as an internal control. Three independent biological replicates were carried out. Similar results were obtained. One of the representative results is shown.


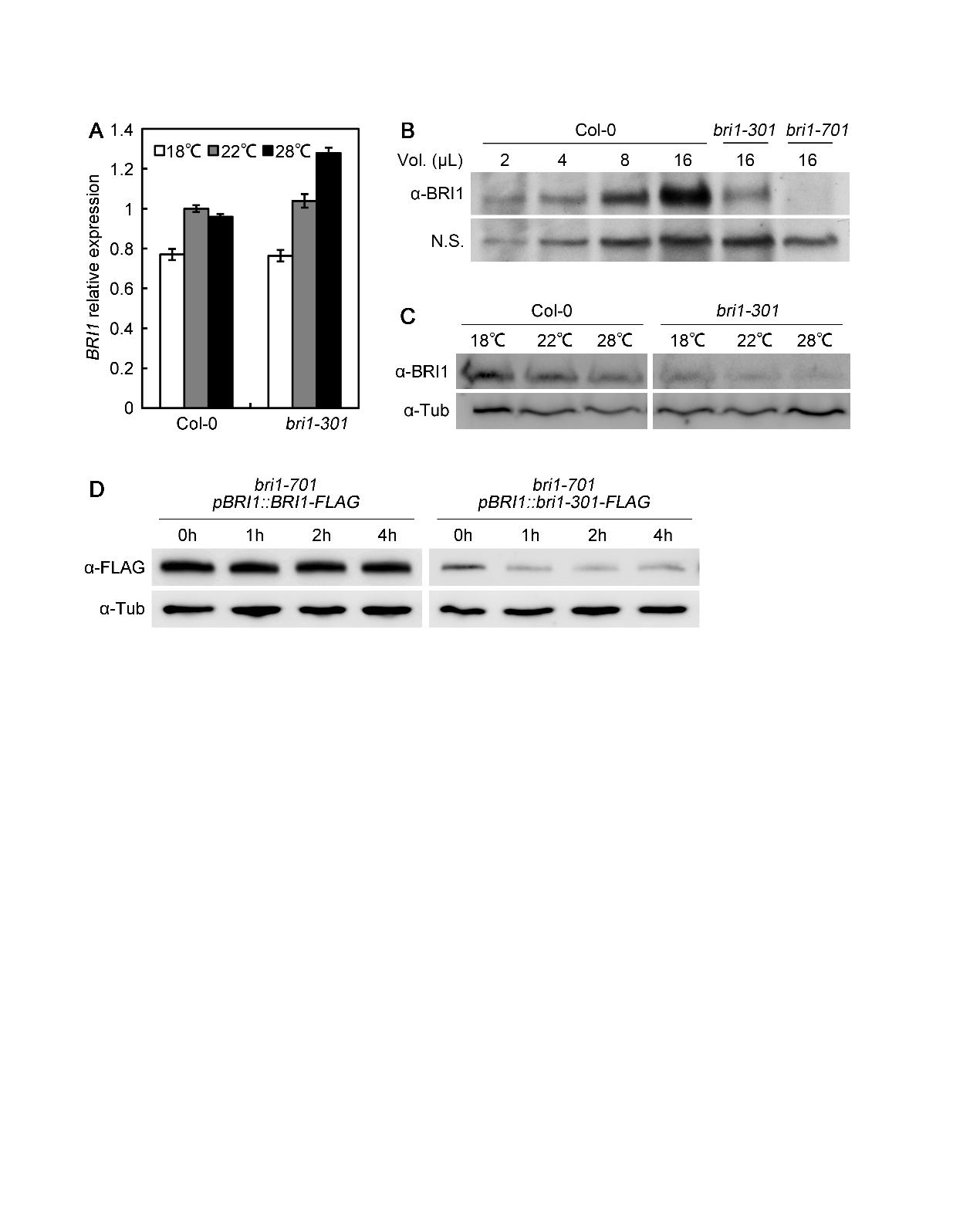


**Figure S4. bri1-301 protein stability is reduced at warmer temperatures.** (**A**) Quantitative RT-PCR results showing *BRI1* expression levels under three different temperature conditions. *ACTIN2* was used as an internal control. (**B and C**) Western blot analyses revealed that the abundance of bri1-301 was dramatically reduced compared with BRI1. *bri1-701* protein extract is used as a negative control. Three independent biological replicates were carried out. Similar results were obtained. One of the representative results is shown.


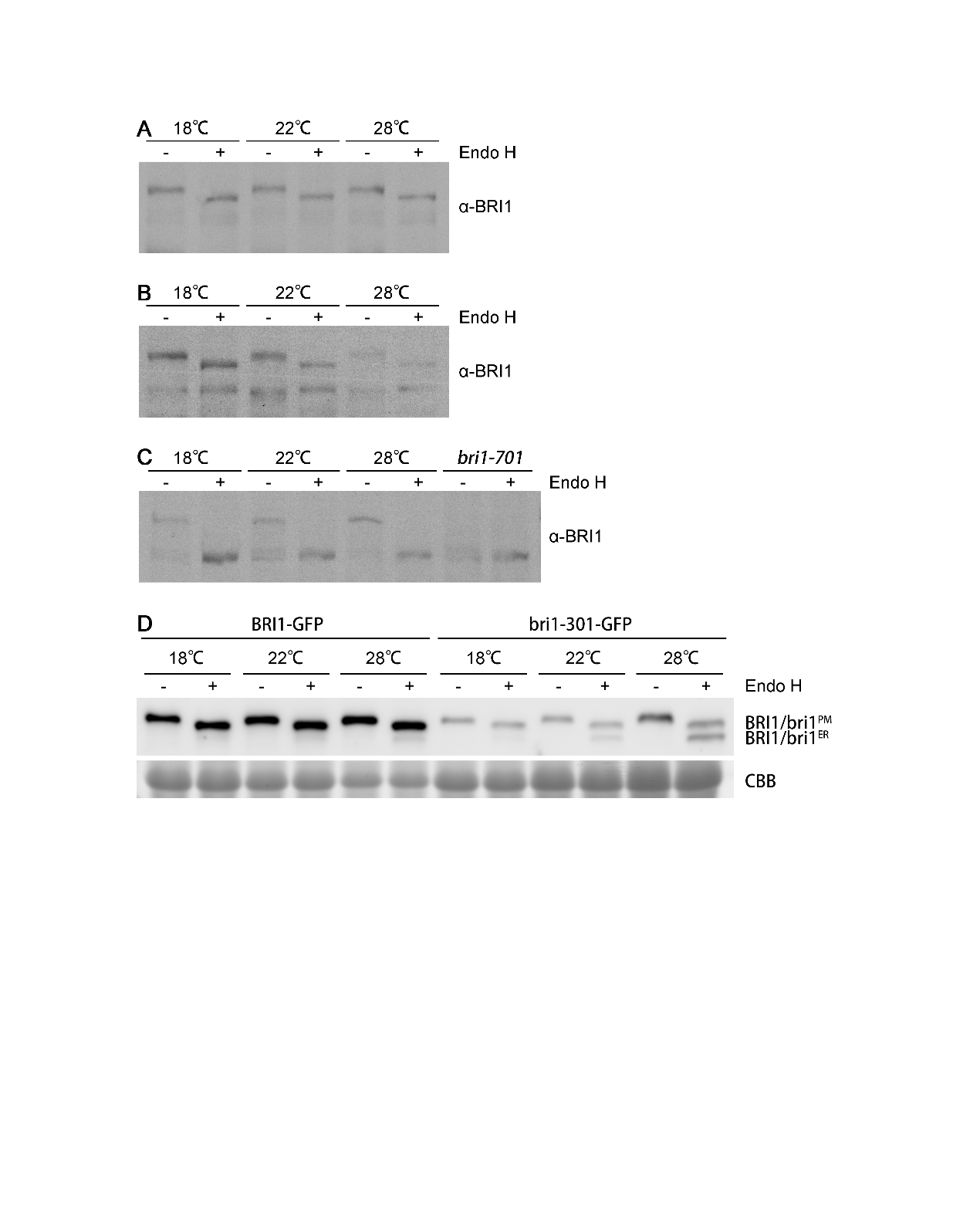


**Figure S5. Endo H assay.** Equal amount of protein samples from 7-day-old seedlings of Col-0 (**A**), *bri1-301* (**B**), *bri1-5* (**C**), and *BRI1/bri1-301-GFP* transgenic seedlings (**D**) were treated with or without Endo H for 1 h at 37°C, followed by Western blot analyses with an anti-BRI1 antibody (**A-C**) and an anti-GFP antibody (**D**), respectively. *bri1-701* was used as a negative control.

**Table S1. Primers used in this research.**

| Used for Genotyping. | |
| --- | --- |
| bri1-301-CAPS-F | AAATCTTGTGCCTCTTCTTGGT |
| bri1-301-CAPS-R | GGGTCAAACACATCGCTAATC |
| bri1-701-GT-F | GATTCCTTTCCTCGGAGATTG |
| bri1-701-GT-R | TTGTTCGGATCTGATTCCTTG |
| LBb1.3 | ATTTTGCCGATTTCGGAAC |
| BRI1-7R | ATCTATTCTCACTTTCCCATTCC |
| Used for quantitative RT-PCR. | |
| ACT2-Q-F | TGTGCCAATCTACGAGGGTTT |
| ACT2-Q-R | TTTCCCGCTCTGCTGTTGT |
| BRI1-Q-F | CTCTCCTGTCTCTCACCGGA |
| BRI1-Q-R | GCACTTGAAGCCAGAAACGG |
| CPD-Q-F | GAGACGCTACGAGTGGCTAA |
| CPD-Q-R | GCATCTTTGAAGTGGTTTGGG |
| SAUR_AC1-Q-F | AGATATGTGGTGCCGGTTTC |
| SAUR_AC1-Q-R | TTGTTAAGCCGCCCATTG |
| Used for gene cloning. | |
| attB1 | GGGGACAAGTTTGTACAAAAAAGCAGGCTTC |
| attB2 | GGGGACCACTTTGTACAAGAAAGCTGGGTC |
| BRI1/bri1-nsc-F | AAAAAGCAGGCTTCATGAAGACTTTTTCAAGCTTCTTT |
| BRI1/bri1-nsc-R | AGAAAGCTGGGTCTAATTTTCCTTCAGGAACTTCTTT |
| G989A-F | CGGAAGATTGCGATAGCATCAGCTAGAGGGCT |
| G989A-R | AGCCCTCTAGCTGATGCTATCGCAATCTTCCG |
| G989C-F | CGGAAGATTGCGATATGTTCAGCTAGAGGGCT |
| G989C-R | AGCCCTCTAGCTGAACATATCGCAATCTTCCG |
| G989D-F | CGGAAGATTGCGATAGATTCAGCTAGAGGGCT |
| G989D-R | AGCCCTCTAGCTGAATCTATCGCAATCTTCCG |
| G989E-F | CGGAAGATTGCGATAGAATCAGCTAGAGGGCT |
| G989E-R | AGCCCTCTAGCTGATTCTATCGCAATCTTCCG |
| G989F-F | CGGAAGATTGCGATATTTTCAGCTAGAGGGCT |
| G989F-R | AGCCCTCTAGCTGAAAATATCGCAATCTTCCG |
| G989H-F | CGGAAGATTGCGATACATTCAGCTAGAGGGCT |
| G989H-R | AGCCCTCTAGCTGAATGTATCGCAATCTTCCG |
| G989I-F | CGGAAGATTGCGATAATATCAGCTAGAGGGCT |
| G989I-R | AGCCCTCTAGCTGATATTATCGCAATCTTCCG |
| G989K-F | CGGAAGATTGCGATAAAATCAGCTAGAGGGCT |
| G989K-R | AGCCCTCTAGCTGATTTTATCGCAATCTTCCG |
| G989L-F | CGGAAGATTGCGATACTATCAGCTAGAGGGCT |
| G989L-R | AGCCCTCTAGCTGATAGTATCGCAATCTTCCG |
| G989M-F | CGGAAGATTGCGATAATGTCAGCTAGAGGGCT |
| G989M-R | AGCCCTCTAGCTGACATTATCGCAATCTTCCG |
| G989N-F | CGGAAGATTGCGATAAATTCAGCTAGAGGGCT |
| G989N-R | AGCCCTCTAGCTGAATTTATCGCAATCTTCCG |
| G989P-F | CGGAAGATTGCGATACCATCAGCTAGAGGGCT |
| G989P-R | AGCCCTCTAGCTGATGGTATCGCAATCTTCCG |
| G989Q-F | CGGAAGATTGCGATACAATCAGCTAGAGGGCT |
| G989Q-R | AGCCCTCTAGCTGATTGTATCGCAATCTTCCG |
| G989R-F | CGGAAGATTGCGATACGATCAGCTAGAGGGCT |
| G989R-R | AGCCCTCTAGCTGATCGTATCGCAATCTTCCG |
| G989S-F | CGGAAGATTGCGATAAGTTCAGCTAGAGGGCT |
| G989S-R | AGCCCTCTAGCTGAACTTATCGCAATCTTCCG |
| G989T-F | CGGAAGATTGCGATAACATCAGCTAGAGGGCT |
| G989T-R | AGCCCTCTAGCTGATGTTATCGCAATCTTCCG |
| G989V-F | CGGAAGATTGCGATAGTATCAGCTAGAGGGCT |
| G989V-R | AGCCCTCTAGCTGATACTATCGCAATCTTCCG |
| G989W-F | CGGAAGATTGCGATATGGTCAGCTAGAGGGCT |
| G989W-R | AGCCCTCTAGCTGACCATATCGCAATCTTCCG |
| G989Y-F | CGGAAGATTGCGATATATTCAGCTAGAGGGCT |
| G989Y-R | AGCCCTCTAGCTGAATATATCGCAATCTTCCG |
